# Peripapillary Retinal Nerve Fiber Measurement with Spectral-Domain Optical Coherence Tomography in Age-Related Macular Degeneration

**DOI:** 10.3390/vision1040026

**Published:** 2017-12-14

**Authors:** Simon K. Law, Kent W. Small, Joseph Caprioli

**Affiliations:** 1 Jules Stein Eye Institute, University of California, Los Angeles, CA 90095–7000, USA; 2 Macula & Retina Institute, Los Angeles, CA 91203, USA

**Keywords:** retinal nerve fiber layer, glaucoma, age-related macular degeneration, optic coherence tomography

## Abstract

*Purpose:* To evaluate the relationship between the peripapillary retinal nerve fiber layer (RNFL) measurements with Spectral-domain Optical Coherence Tomography (OCT) and Age-related macular degeneration (AMD). *Methods:* Patients >60 years of age without glaucoma or record of intraocular pressure >21 mmHg and no systemic or intraocular diseases or treatment or surgical intervention that affected the RNFL underwent OCT measurement of the RNFL. The severity of AMD was staged with the Clinical Age-Related Maculopathy Staging System. The relationship between RNFL measurements and AMD stages of one eye per patient was analyzed. *Results:* Eighty-six eyes (46 patients) with AMD and no glaucoma or other exclusion criteria received OCT RNFL measurements. Nine eyes (10.5%) were excluded because of distorted peripapillary anatomy from exudative AMD (7 eyes) or failure of the RNFL segmentation algorithm (2 eyes). Mean age ± S.D. of the 43 patients analyzed was 81.2 ± 7.3 years. The mean stage ± S.D. of AMD of the 77 eyes was 3.77 ± 1.05. Higher stages of AMD were statistically significantly associated with lower average RNFL and inferior sector RNFL (*p* = 0.049, 0 0015, respectively). The association of inferior sector RNFL and AMD stage remained statistically significant after adjusting for age. *Conclusions:* Spectral domain OCT is generally useful in measuring the peripapillary RNFL in eyes with different stages of AMD. Higher stage of AMD is associated with thinner peripapillary RNFL, which may masquerade as early glaucomatous damage.

## 1. Introduction

Age-related macular degeneration (AMD) and glaucoma are the two leading causes of irreversible blindness in the United States [1,2]. Based on the pooled prevalence data predicting that a Caucasian-American woman age 80 years or older has a 16.4% likelihood of having AMD and a 7.0% likelihood of having glaucoma, Alward expected that 7.0% of elderly white women with AMD would also have glaucoma, assuming that AMD and glaucoma are two independent diseases [3]. However, recent research suggests a possible comorbid association of these two diseases [4,5]. In a retrospective case-control study on Medicare beneficiaries, patients with exudative AMD are significantly more likely to have comorbidities than individuals without AMD. More than 60% greater odds of glaucoma were identified in the exudative AMD cohort [4]. In another study evaluating Medicare claims longitudinally over 9 years, Lee et al. reported about one third of patients with glaucoma also had AMD and vice versa [5].

In a previous retrospective comparison of fellow eyes of patients with asymmetric stages of AMD with quantitative optic disc tomographic measurements with confocal laser ophthalmoscopy (Heidelberg Retinal Tomography; HRT), we demonstrated that in eyes with extensive advanced stages of macular degeneration, the structural appearance of the optic disc is more glaucoma-like [6]. However, in reviewing the previous data obtained for the Beaver Dam Eye Study to evaluate the relationship between AMD and clinical optic disc appearance, Hall and associates did not find any significant difference in the cup size, disc size and cup/disc ratio among groups of eyes with no AMD, early AMD and late AMD. In addition, no significant relationship was found between size of the area involved by lesion of AMD and the clinical cup/disc ratio in eyes with late AMD [7].

Optical coherence tomography (OCT) is a high-resolution imaging technique based on the optical principle of low-coherence interferometry. It is capable of producing objective and reproducible cross-sectional images and measurements of optic disc and retinal nerve fiber layer (RNFL) structure [8,9,10]. The early OCT is a time-domain OCT imaging where A-scan tissue-reflectance information is obtained over time by moving a mirror in the reference arm of the interferometer and OCT B-scans are generated by acquiring several neighboring A-scans. The newer generation of OCT is the Spectral-domain OCT which can acquire entire A-scans in one instance by measuring frequency components of reflected light at a given point in tissue. Information on depth is transformed from the frequency domain to the time domain and a moving reference mirror is not necessary. As a result, Spectral-domain OCT can obtain images much faster, minimize the effect of ocular movements and reduce the image’s noise level. The quality of the scan with Spectral-domain OCT is superior to time-domain OCT [10]. Recent research on Spectral-domain OCT showed good agreement with time-domain OCT and both OCT technologies performed well to diagnose glaucoma [11,12,13,14]. Using Spectral-domain OCT to measure the peripapillary RNFL thickness in Japanese patients with unilateral exudative AMD, Yuda and associates found that RNFL thickness in eyes with exudative AMD did not differ significantly from the fellow eyes free of exudative AMD [15]. However, 63% of the population sample had exudative macular changes from the AMD subtype of polypoidal choroidal vasculopathy, which is a more common form of AMD in the Japanese patients and may have less exudative change than in typical AMD. Authors cautioned that possible genetic differences between the Japanese population and Caucasian population may affect the phenotypic presentation of AMD in the two populations [15]. It is uncertain if similar results apply to AMD patients of other ethnicities. Additional research in the relationship between the structure of the optic disc or RNFL and AMD is necessary.

In this study, we prospectively measured the peripapillary RNFL with Spectral-domain OCT in patients with AMD and evaluated the relationship between the RNFL measurements and severity of AMD classified by a standardized clinical staging system. We hypothesized that in patients with AMD, the severity of AMD correlates with the peripapillary RNFL measurements with Spectral-domain OCT.

## 2. Methods

The study protocol was approved by the Institutional Review Board of the University of California, Los Angeles. Patients who had AMD and were 60 years of age or older that did not have glaucoma, or did not have any record of intraocular pressure (IOP) more than 21 mmHg or visual field defects that were suspected to be glaucomatous were invited to participate in the study. Patients were excluded if they had ocular or neurological diseases affecting the retinal nerve fiber layer, history of ocular laser treatment or intraocular injection of medication for AMD (including anti-vascular endothelial growth factor (anti-VEGF) agents and steroids), or intraocular surgery other than uncomplicated cataract surgery. Dilated fundus examination was performed. The severity of AMD was staged with the Clinical Age-Related Maculopathy Staging System (CARMDS) [16]. This grading system classifies AMD into 1 of 5 mutually exclusive stages (Table 1). The various classes of AMD are estimated based on the presence, size and approximate number of drusen; presence of retinal pigment epithelial hypopigmentation or hyperpigmentation; drusenoid or serous retinal pigment epithelial detachment; geographic atrophy; choroidal neovascularization; and sub retinal scarring. The enrollment of patients with AMD was not limited to a particular stage of AMD.

The thickness of the peripapillary RNFL was measured with Spectral OCT SLO Combination Imaging System^®^ (OPKO Health, Inc., Miami, FL, USA). The basic principles of the OCT have been well described in the literature [17]. A circular OCT scan with a diameter of 3.4 mm is placed around the optic disc while the location is observed on the confocal scanning laser ophthalmoscopy image to ensure proper positioning in relation to the optic disc. Three measurements were performed for each eye for averaging. The quality of Spectral-domain OCT RNFL images was inspected individually. Images with distorted anatomy of the peripapillary RNFL secondary to exudative AMD or the RNFL segmentation algorithm failed to identify the RNFL were excluded.

The following OCT parameters were used for analyses: average RNFL thickness and mean RNFL thickness in each of eight 45-degree sectors. Sectors were defined as follows: superior, superior-temporal, temporal, inferior-temporal, inferior, inferior-nasal, nasal and superior-nasal sectors.

Statistical analyses were performed with SPSS statistical software, version 16.0, for Windows (SPSS Inc., Chicago, IL, USA). The relationships between RNFL measurements and AMD stages and between RNFL measurements and age were analyzed statistically with a logistic regression model. Only one eye per patient was used for this analysis. If both eyes were enrolled and the quality of images was acceptable, the right eye was arbitrarily selected for analysis. Statistical significance was defined as *p* < 0.05 for average RNFL measurement. Bonferroni correction was applied for multiple comparisons of sectoral RNFL thickness.

## 3. Results

Between November 2007 and February 2009 (15 months), a total of 86 eyes (46 patients) with AMD and no glaucoma or other exclusion criteria had measurements of the peripapillary RNFL by Spectral-domain OCT. Nine eyes of 8 patients (10.5% by eyes) were excluded because of distorted peripapillary anatomy from exudative AMD (7 eyes) or the RNFL segmentation algorithm failed to identify a portion of the RNFL (2 eyes). The RNFL measurements of 77 eyes (43 patients) were analyzed.

Mean age ± standard deviation (S.D.) of the 43 patients was 81.2 ± 7.3 years and 55.8% of patients were female. The mean stage ± S.D. of AMD of the 77 eyes was 3.77 ± 1.05. There were no eyes in stage 1, 8 eyes in stage 2 (10.4%), 29 eyes in stage 3 (37.7%), 13 eyes in stage 4 (16.9%) and 27 eyes in stage 5 (35.1%).

**Figure 1:**
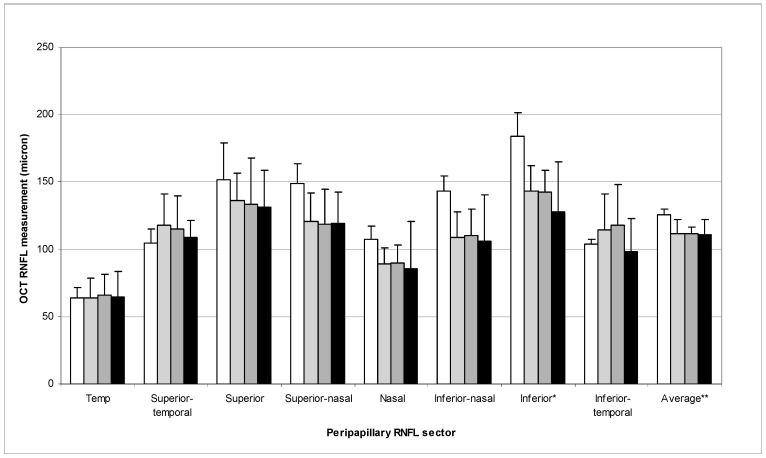
RNFL measurements of different peripapillary sectors according to AMD stages (white bar: stage 2, light gray bar: stage 3, dark gray bar: stage 4, black bar: stage 5). Data are presented as mean ± standard error of the mean. (OCT: Optical coherence tomography; AMD: Age-related macular degeneration; RNFL: Retinal nerve fiber layer. * *p* = 0.015; ** *p* = 0.049).

**Figure 2:**
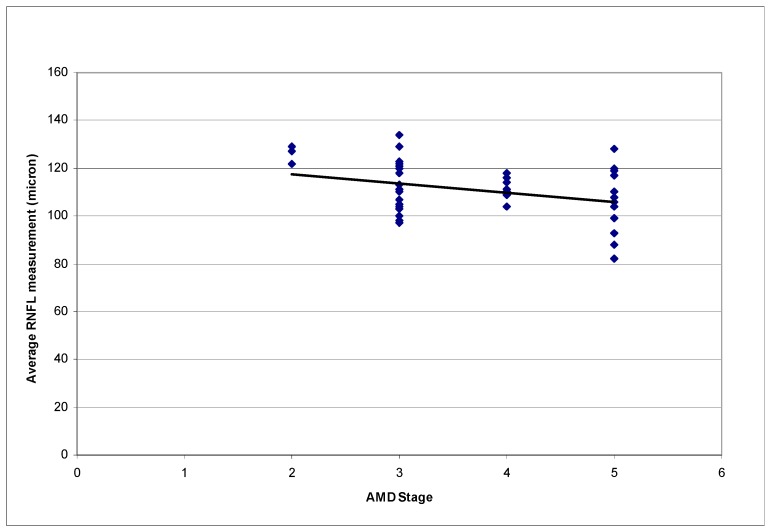
Correlation between Average RNFL measurements by Spectral-domain OCT and AMD stages. (*p* = 0.049, R^2^ = 0.117; OCT: Optical coherence tomography; AMD: Age-related macular degeneration; RNFL: Retinal nerve fiber layer.).

**Figure 3:**
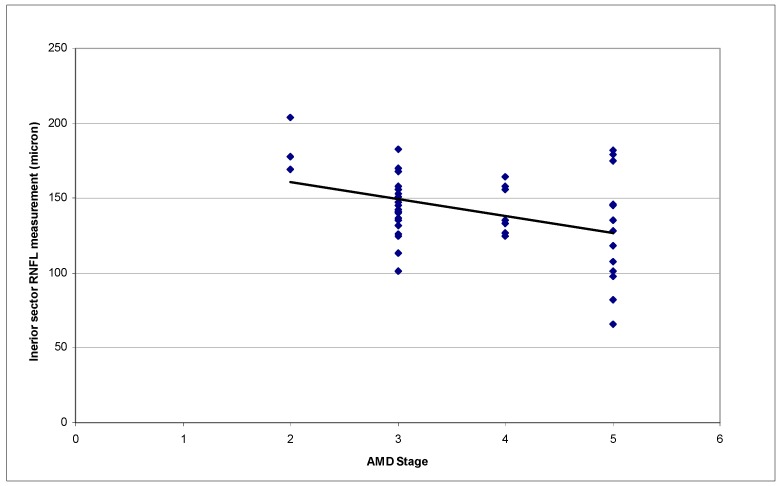
Correlation between inferior sector RNFL measurements by Spectral-domain OCT and AMD stages. (*p* = 0.015, R^2^ = 0.157; OCT: Optical coherence tomography; AMD: Age-related macular degeneration; RNFL: Retinal nerve fiber layer.).

In assessing relationships between RNFL measurements and AMD stages and between RNFL measurements and age, only one eye per patient was used for analysis. If both eyes had AMD and were enrolled, the right eye was arbitrarily selected. The RNFL measurements of 43 eyes (43 patients) were analyzed. Table 2 summarizes the mean RNFL measurements of the 43 eyes. Figure 1 represents the RNFL measurements of different peripapillary sectors according to AMD stages. Statistically significant correlations were found between the average RNFL measurements and AMD stages (*p* = 0.049, R^2^ = 0.117). A higher AMD stage was associated with a thinner RNFL (Figure 2 and Figure 3). The correlation between inferior sector RNFL measurements and AMD stages was not statistically significant after Bonferroni correction (*p* = 0.015, R^2^ = 0.157, *p*-value after Bonferroni correction = 0.12).

**Figure 4:**
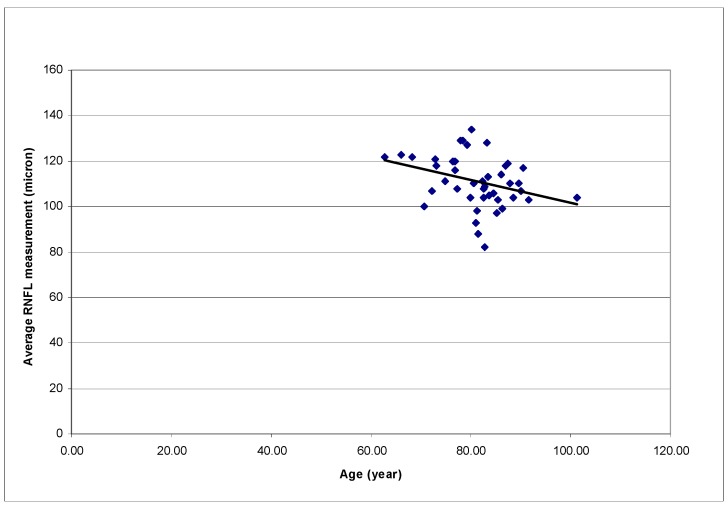
Correlation between Average RNFL measurements by Spectral-domain OCT and patients’ age. (*p* = 0.033, R^2^ = 0.106; OCT: Optical coherence tomography; AMD: Age-related macular degeneration; RNFL: Retinal nerve fiber layer.).

Patient age was also found to be statistically significantly correlated with the average RNFL measurement and the RNFL measurements at the superior temporal sector (*p* = 0.033, R^2^ = 0.106; *p* = 0.037, R^2^ = 0.102; respectively) Older age was associated with a thinner RNFL (Figure 4). When both age and AMD stage were entered into a multivariate model for analysis, only AMD stage remained statistically significantly correlated with the RNFL thickness of the inferior sector (*p* = 0.040).

## 4. Discussions

In this prospective analysis of peripapillary RNFL measurements obtained with Spectral-domain OCT, the thickness of the peripapillary RNFL had a small but statistically significant correlation with the severity of AMD classified according to the Clinical Age-Related Maculopathy Staging System (CARMDS). Higher AMD stages were associated with a thinner average peripapillary RNFL and possibly with the RNFL at the inferior sector.

Previous analysis of the optic disc in eyes with AMD with confocal ophthalmoscopy (Heidelberg Retinal Tomography; HRT) and clinical optic disc evaluation have showed that larger areas of macular damage of advanced AMD has a statistically significant association with optic disc of smaller rim area and larger cup/disc ratio and more likely to be classified as glaucomatous [6]. However, the retrospective design of this study has the limitation of possible selection bias of including patients with existing glaucoma. In the current study, we prospectively excluded patients with any ocular history of glaucoma, elevation of IOP, or visual field defects that were suspected to be glaucomatous.

The current result is in contrast to the findings of the previous study by Yuda et al. using Spectral-OCT to compare the peripapillary RNFL thickness between the eyes with exudative AMD and the fellow eyes without exudative AMD in Japanese patients [15]. In the Yuda’s study, peripapillary RNFL thickness in the eyes with exudative AMD did not differ significantly from the fellow eyes. However, the severity of AMD of the fellow eyes without exudative AMD was not reported. Their report also found no association between the lesion’s size and the peripapillary RNFL thickness in contrast to our previous report using HRT to compare the optic disc parameters of the fellow eyes with asymmetric involvement of advanced AMD [6]. The differences between the two reports may be partially explained by the difference in the size of the lesion included in analysis. In the Yuda’s study, the mean ± S.D. of lesion size was 5.06 ± 4.69 disc areas according to the MPS system; and in our previous study, statistically significant correlation between the HRT measurements of the optic disc and AMD was found only in eyes with six or more disc areas of macular lesion of AMD. In the current study, the association between the lesion’s size and the peripapillary RNFL thickness was not investigated since the lesion’s size could not be estimated in all eyes with only approximately half (52%) of this sample had geographic atrophy or exudative AMD.

The two imaging techniques (HRT and OCT) measure different parameters of the optic disc and may not be directly comparable. The HRT analyzes the contour of the optic disc and the RNFL scan of OCT measures the thickness of the peripapillary RNFL. There is also a difference in the classification system used to estimate the severity of AMD in different studies. Previous studies focused on the extent of advanced AMD defined as geographic atrophy or disciform scar, which was determined with a disc-area template developed for the Macular Photocoagulation Study (MPS) [18]. The Clinical Age-Related Maculopathy Staging System (CARMS) was used in the current study. It has been validated and proven to be a reliable staging system that can be used in both clinical practice and in clinical research protocols involving patients with AMD [15].

In the current study, the peripapillary RNFL thinning at the inferior sector correlated with higher stages of AMD, though not statistically significant after Bonferroni correction was applied. The inferior rim of the optic disc tends to be the area of early damage of glaucomatous optic neuropathy [19,20]. Thinning of the peripapillary RNFL at the inferior sector may represent an early glaucomatous optic neuropathy. However, a large area of macular degeneration may involve the RNFL that occupy the inferior sector of the optic disc and the thinning of RNFL at this sector may be misinterpreted as glaucomatous damage.

In reviewing the Spectral-domain OCT RNFL images of the patients enrolled in this study, despite our best efforts to capture good quality OCT images, approximately 10% (9 of 86 eyes) were excluded because of distorted peripapillary anatomy from exudative AMD, or the RNFL segmentation algorithm failed to identify a portion of the RNFL.

This study has limitations of a relatively small sample size and a lack of longitudinal follow-up of the RNFL measurements. Since the beginning of patient recruitment in late 2007, antiangiogenic therapy with anti-VEGF has become a common treatment modality for advanced AMD [21]. Intravitreal injection of anti-VEGF agents has been associated with sustained elevation of IOP in some patients with AMD [22,23,24]. Patients of this study had the peripapillary RNFL measurements obtained before any intravitreal injection of medications.

Despite the popularity of intravitreal injection of anti-VEGF agents as a treatment for AMD, research on the effect of anti-VEGF agents on the optic disc is limited. Horsley and associates retrospectively compared the time-domain OCT (Stratus OCT, Carl Zeiss Meditec, Dublin, California, USA) peripapillary RNFL measurements before and after multiple intravitreal injections of anti-VEGF agents in 41 eyes of 37 consecutive patients that had greater than 10 total anti-VEGF injections. The average ± S.D. RNFL thickness at presentation was 92.4 ± 15.2 μm and at last follow-up was 93.8 ± 15.2 μm (*p* = 0.68). There were also no statistically significant differences in RNFL measurements when comparing between individual anti-VEGF agents [25]. In another retrospective study, Seth and associates also found no statistically significant change in the vertical cup/disc ratio in patients receiving multiple intravitreal injections of anti-VEGF agents [26]. Prospective longitudinal studies are needed to verify the finding of these studies. If it is verified that intravitreal anti-VEGF therapy is not associated with significant change of the optic disc or RNFL, OCT may be a useful tool in following the progression of the RNFL for glaucoma detection.

Evaluation for glaucoma in individuals with AMD is more challenging than in patients without AMD. Evaluation of glaucoma primarily relies on IOP measurement, visual field testing and optic nerve evaluation. However, IOP may not be elevated in glaucoma, visual field defects associated with the maculopathy from AMD may be difficult to differentiate from those associated with glaucomatous optic neuropathy and the large variation in the structural appearance of a normal optic disc optic nerve greatly reduces the certainty of the optic disc evaluation. Newer digital technologies that can provide quantitative evaluation of the structural appearance of the optic disc and RNFL have shown able to help detect early glaucoma or even the progression of the disease. The findings of this study suggest that the reduction in average RNFL measurement with advancing AMD is minimal and Spectral-domain OCT is generally useful in measuring the peripapillary RNFL in eyes with different stages of AMD. However, the distortion of the peripapillary RNFL associated with AMD observed in some eyes with exudative changes may preclude the application of OCT in the evaluation of RNFL in a small percentage of patients. In addition, eyes with higher stages of AMD may have a thinner peripapillary RNFL and more research in the relationship between AMD and glaucoma, two major ocular diseases, is necessary.

## Figures and Tables

**Table 1:** The Clinical Age-Related Maculopathy Staging (CARMS) System.

Grade of Maculopathy	Clinical Features
1	No drusen or <10 small drusen without pigment abnormalities
2	Approximately ≥10 small drusen or <15 intermediate drusen, or pigment abnormalities associated with ARM
	a. Drusen
	b. RPE changes (hyperpigmentation and hypopigmentation)
	c. Both drusen and RPE changes
3	Approximately ≥15 intermediate drusen or any large drusen
	a. No drusenoid RPED
	b. Drusenoid RPED
4	Geographic atrophy with involvement of the macular center, or non-central geographic atrophy at least 350 μm in size
5	Exudative AMD, including non-drusenoid pigment epithelial detachments, serous or hemorrhagic retinal detachments, CNVM with sub retinal or sub-RPE hemorrhages or fibrosis, or scars consistent with treatment of AMD
	a. Serous RPED, without CNVM
	b. CNVM or disciform scar

RPE: Retinal pigment epithelium; AMD: Age-related macular degeneration; CNVM: Choroidal neovascular membrane; RPED: Retinal pigment epithelium detachment.

**Table 2:** Mean peripapillary RNFL measurements by Spectral-domain OCT of the 43 eyes (43 patients).

Peripapillary Sector	RNFL Measurement (Mean ± S.D. in Micron)
Temporal	64.53 ± 15.28
Superior Temporal	113.81 ± 22.72
Superior	135.40 ± 25.08
Superior Nasal	122.12 ± 22.76
Nasal	89.37 ± 21.78
Inferior Nasal	110.77 ± 25.41
Inferior	141.28 ± 28.27
Inferior Temporal	109.40 ± 26.61
Average	110.95 ± 11.31

OCT: Optical coherence tomography; RNFL: Retinal nerve fiber layer.
